# A Case Report on Medial Patellofemoral Ligament Reconstruction Rehabilitation in a Cricket Player Through Targeted Agility Training

**DOI:** 10.7759/cureus.56002

**Published:** 2024-03-12

**Authors:** Ashish Keoliya, Swapnil U Ramteke

**Affiliations:** 1 Sports Physiotherapy, Ravi Nair Physiotherapy College, Datta Meghe Institute of Higher Education and Research, Wardha, IND

**Keywords:** adolescent sports injuries, athletic rehabilitation, patellar instability, agility training, medial patellofemoral ligament reconstruction

## Abstract

The medial patellofemoral ligament (MPFL) serves as a crucial stabilizer, preventing lateral dislocation of the patella. Surgery is usually advised after a second dislocation, with MPFL reconstruction being a common procedure. The primary objective of rehabilitation post-surgery is not just to relieve pain and restore range of motion (ROM), but also to combine proprioceptive neuromuscular training to help patients return to their prior functional level. Agility training is a crucial component in accomplishing this goal. The current case is of a 19-year-old male cricket player with a history of recurrent patellar dislocation who underwent surgery after the latest incident rendered him unable to bear weight. His rehabilitation program included standard protocols alongside the early implementation of agility drills, such as ladder exercises. Compared to traditional approaches, this case demonstrates significant improvement in the patient's functional ability following surgery. This report highlights the importance of comprehensive rehabilitation for patients undergoing MPFL reconstruction. It not only underscores the rationale behind the surgery but also lays the groundwork for comparing recovery times. Notably, this program incorporated early agility exercises from the very beginning, suggesting that such an approach might accelerate recovery compared to standard protocols. Further investigation is warranted to explore the potential benefits of early agility training in this patient population.

## Introduction

Instability in the knee, such as patellar dislocation and patellofemoral instability, can impact individuals in their youth irrespective of their level of physical activity. Unfortunately, once someone experiences a patellar dislocation, they have a high chance of it happening again. In fact, nearly 40% of people experience a repeat patellofemoral joint dislocation after the initial event. This risk is even higher after a repeat dislocation, with almost half (49%) of patients experiencing another one [1]. Unlike anterior cruciate ligament (ACL) and posterior cruciate ligament (PCL) tears, common sports injuries typically occur when the knee experiences excessive forward (anterior) or backward (posterior) movement of the shinbone (tibia) relative to the thighbone [2]. MCL tears typically happen when the knee is forced inward while planted, stretching the ligament beyond its limits [3]. This contrasts with medial patellofemoral ligament (MPFL) tears, which occur when the kneecap dislocates sideways. Interestingly, having both an MPFL tear and an ACL or MCL tear at the same time is quite rare. MPFL injuries are not limited to one sport, like cricket. Athletes can experience them during a variety of activities that require knee movement, turning, and fast directional changes. Young athletes, in particular, frequently face the difficulty of patellar instability; nearly half (51.9%) of patellar dislocations occur during sporting activities, with basketball (18.2%), soccer (6.9%), and football (6.3%) accounting for the highest frequency of patellar dislocations [4].

The MPFL is a crucial stabilizing ligament around the patella, which plays a key role in resisting lateral migration and maintaining central alignment within the patellofemoral groove [5,6]. Located within the second layer of tissues on the knee's inner side, this distinct ligament plays a critical role in knee stability. The MPFL originates from a triangular area between two bony structures, i.e., adductor tubercle and medial epicondyle, and attaches to the upper two-thirds of the kneecap's inner edge. Measuring roughly 55 mm long and varying in width (3-30 mm), it can withstand significant forces of approximately 203 N on average. Interestingly, this ligament is tight when the knee is fully extended, and the quadriceps muscle is engaged. The medial patellotibial ligament (MPTL) and medial patellomeniscal ligament (MPML) play a supporting role in stabilizing the kneecap (patella) along with the main stabilizer, the vastus medialis oblique (VMO) muscle. While the MPTL and MPML are important for medial patellar stability, their influence becomes less significant as knee flexion increases beyond 30°. At this point, the vast medial oblique muscle takes over as the primary stabilizer, ensuring the kneecap tracks properly within the joint [7]. When it comes to patellar instability (kneecap dislocation), conservative treatment is always the first line of defense. Conservative management typically involves rehabilitation focusing on the pelvis and upper leg, progressive strengthening exercises specifically targeting the VMO muscle (located on the inner thigh), and the use of supportive braces [8].

A first-time kneecap dislocation often damages the MPFL significantly, with nearly all patients (around 96%) potentially requiring surgical reconstruction to restore stability. MPFL reconstruction is a common procedure, especially when bony structures are within the normal or near-normal range, often performed independently without the need for additional realignment like trochleoplasty or tibial tuberosity osteotomy [9]. Selecting the right graft for ligament reconstruction hinges critically on several mechanical factors, including tendon tensile strength and viscoelastic properties. Despite the absence of direct clinical comparisons in the current literature, research has explored the biomechanical characteristics of various potential grafts. According to Mountney et al., the MPFL ruptures at an average of 26±7 mm. This rupture point roughly coincides with patellar dislocation occurring around 50 mm, suggesting that MPFL failure precedes the dislocation itself [10,11].

MPFL reconstruction emerges as an effective and reliable treatment for patellofemoral instability, offering a high probability of patients returning to sports, either alone or in conjunction with osteotomy. The procedure is deemed safe and successful for individuals without severe trochlear dysplasia, allowing most to resume regular sports activities at a recreational level within two years post-operatively. However, surgeons must exercise caution due to potential complications such as persistent instability, pain, and loss of flexion [12,13].

This case report delves into the rehabilitation strategy for a young cricket player who underwent MPFL reconstruction using a semitendinosus tendon autograft coupled with a tuberosity osteotomy. Since knee dislocation injuries vary, rehabilitation plans need to be individualized. This case explores incorporating agility ladder training into the recovery process. Despite the increasing prevalence of this diagnosis and surgical procedure, there remains a dearth of research guiding the clinical rehabilitation process post-operation. Furthermore, more studies are needed to identify appropriate outcome measures to assess treatment efficacy for this specific patient population.

## Case presentation

Patient information

**Figure 1:**
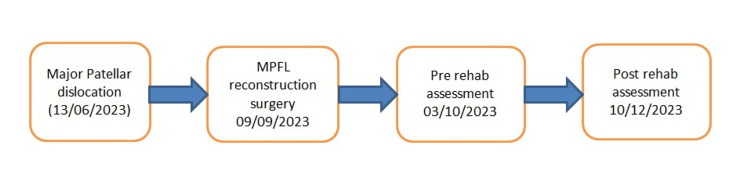
Figure showing the sequence of events

This case report features a 19-year-old male cricket player who sought treatment at the sports physical therapy department after undergoing MPFL reconstruction. The patient was a college student actively engaged in cricket and reported a history of patellar dislocation in the right knee during gameplay, resulting in immediate resolution (June 13, 2023). Subsequently, he experienced swelling and pain in the affected knee. The patient initially sought medical attention from a local practitioner. The local practitioner prescribed pain medication and advised rest. As the pain diminished, the patient resumed playing until experiencing another dislocation, leading to his referral to the orthopedic department at Acharya Vinoba Bhave Rural Hospital (AVBRH), Sawangi. Radiological investigation revealed a complete rupture of the MPFL. Consequently, it was decided that the individual should undergo MPFL reconstruction surgery. He got operated on September 9, 2023. The patient was given a post-operative partial-weight-bearing (WBAT) status along with home-based exercises (mentioned in phase 1 of the physiotherapy management table) for four weeks with the use of a walker and a locked knee extension brace. On October 10, 2023, the patient visited the sports physiotherapy department after undergoing surgery, reporting a chief complaint of dull pain in the right knee. The pain was noted during activities such as knee flexion, prolonged sitting, stair negotiation, and pressure to the patella. This discomfort had persisted for approximately four months since a sport-related patellar dislocation episode. After a comprehensive analysis, a plan was made to fix the problems we found during the assessment. Figure 1 shows the sequence of events in graphical format.

Clinical findings

**Figure 2:**
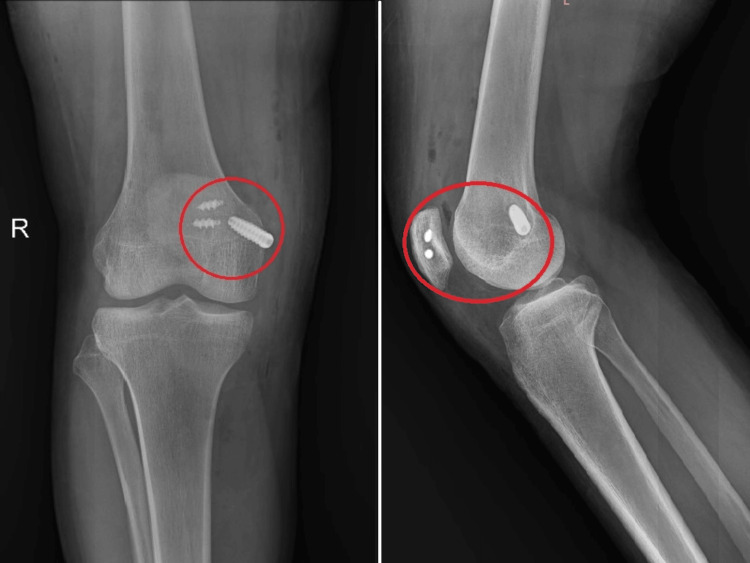
Post-operative X-ray showing patellar suture anchors and interference screw at the Schöttle point of the femur Schottle's point serves as a radiographic reference indicating the isometric point for femoral insertion in the context of MPFL reconstruction. MPFL, medial patellofemoral ligament

The physical examination revealed two minor incisions around the kneecap, each about 1 cm long. There was also a 2-inch incision on the inner side for drilling, a 1-inch cut near the inner thigh for drilling, and a 1-inch cut on the outer thighs. The swelling was visible beneath the kneecap and pressing on it caused considerable pain. The participant had slightly decreased sensation in the right inner knee area. Reflexes were typical in both legs, with the exception of the right kneecap reflex, which was somewhat weaker. The individual walked with a limp, taking shorter steps on the right side and placing less weight on the left leg. Utilizing this evaluation, a proficient treatment strategy was formulated, integrating physical therapy to alleviate pain and enhance muscular strength. Given his identity as a cricket athlete, agility training was also introduced in the later stages to assist him in reclaiming his pre-injury status. Figure 2 shows patients post-operative X-rays.

Findings such as lower extremity (LE) goniometric range of motion (ROM) are presented in Table 1.

**Table 1:** LE goniometric ROM in degrees ROM is calculated in degrees (^o^). Normal ranges for hip: flexion (0^o^-135^o^), extension (0^o^-30^o^), abduction (0^o^-45^o^), and adduction (0^o^-20^o^). Normal ranges for the knee: flexion (0^o^-150^o^) and extension (0^o^) ROM, range of motion; LE, lower extremity

Motion (right side)	Pre-rehabilitation	Post-rehabilitation
Hip flexion	100^o^	130^o^
Hip extension	10^o^	15^o^
Hip abduction	30^o^	45^o^
Hip adduction	10^o^	20^o^
Knee flexion	80^o^	120^o^
Knee extension lag	10^o^	0^o^

Manual muscle testing values of lower extremity (LE) pre- and post-rehabilitation are presented in Table 2.

**Table 2:** LE manual muscle testing MRC scale for muscle strength: grade 0: no visible contraction, grade 1: visible contraction without movement of the limb, grade 2: movement of the limb but not against gravity, grade 3: the movement against gravity over, grade 4: the movement against gravity and resistance, and grade 5: normal. *With consistent pain LE, lower extremity; MRC, Medical Research Council

Motion	Right LE (pre-rehabilitation)	Right LE (post-rehabilitation)
Flexion	3	5
Hip extension	3	4
Hip abduction	3^*^	5
Hip adduction	3	4
Hip external rotation	3	4
Hip internal rotation	3	4
Knee flexion	3^*^	5
Knee extension	3^*^	5

Physiotherapy management

The rehabilitation of MPFL reconstruction surgery involves a phased approach. The initial weeks (phase 1) focus on protecting the surgical site with minimal movement, using tools like ice and braces. Gentle exercises target swelling control and basic muscle activation. As healing progresses (phase 2), therapists introduce more movement exercises and light strengthening to combat muscle weakness and stiffness. Balance and coordination training also begin. Finally, phase 3 emphasizes regaining a full, pain-free ROM, building lower limb (LL) strength and endurance, and incorporating sport-specific activities into your exercise routine. This progressive program, guided by a physiotherapist, helps you return to your desired level of activity. The detailed management is given below in Table 3.

**Table 3:** Physiotherapy management ROM, range of motion; LL, lower limb; VMO, vastus medialis oblique; CKC, closed kinetic chain; NMES, neuromuscular electrical stimulation; NWB, non-weight-bearing; PWB, partial weight-bearing

Phase	Goal of phase	Physiotherapy intervention	Exercise program	Functional/sports-specific activity
Phase 1: Maximum protection phase, weeks 0-4 (mostly prescribed as a home exercise program except for modalities at the time of discharge)	Protect surgical site and soft tissue healing. Control swelling. prevent post-operative complications	Cryotherapy with elevation to avoid swelling. Knee locked in extension with long knee brace while ambulation	Passive ROM and grades I and II superior and inferior patellar glides. Static quadriceps and hamstring exercises. Active ankle-toe movements	Only NWB or PWB with knee locked-in extension
Phase 2: Protected motion phase, weeks 4-7 (actual rehabilitation started at sports physiotherapy department)	Reduce pain and swelling and avoid contractures. Avoid quadriceps lag and initiate LL and core muscle activation	Interferential electrical current and ice for pain and swelling. Quad sets with NMES for VMO activation.	Knee isometrics for extension. Initiate straight leg raises, clamshells, fire hydrants, and hamstring curls. Unilateral LL proprioceptive exercises in a locked brace.	PWB, progress to FWB walking (achieve normal gait pattern). Agility ladder exercises with unaffected limb
Phase 3: Motion and muscle activation phase, weeks 7-12	Achieve pain-free ROM. Increase LL endurance. Increase LL strength. Proper functioning of the extensor mechanism. Increase flexion	CKC exercises. Balance and proprioception exercises with a balance board or agility ladder	Progression of the ROM exercises for LL single-leg balancing exercises without braces. Wall slides for knee flexion, progressing to mini-squats. Hamstring curls functional progression, sport-specific activities	Walking programs. Stationary cycle, agility ladder exercises with affected limb

**Figure 3:**
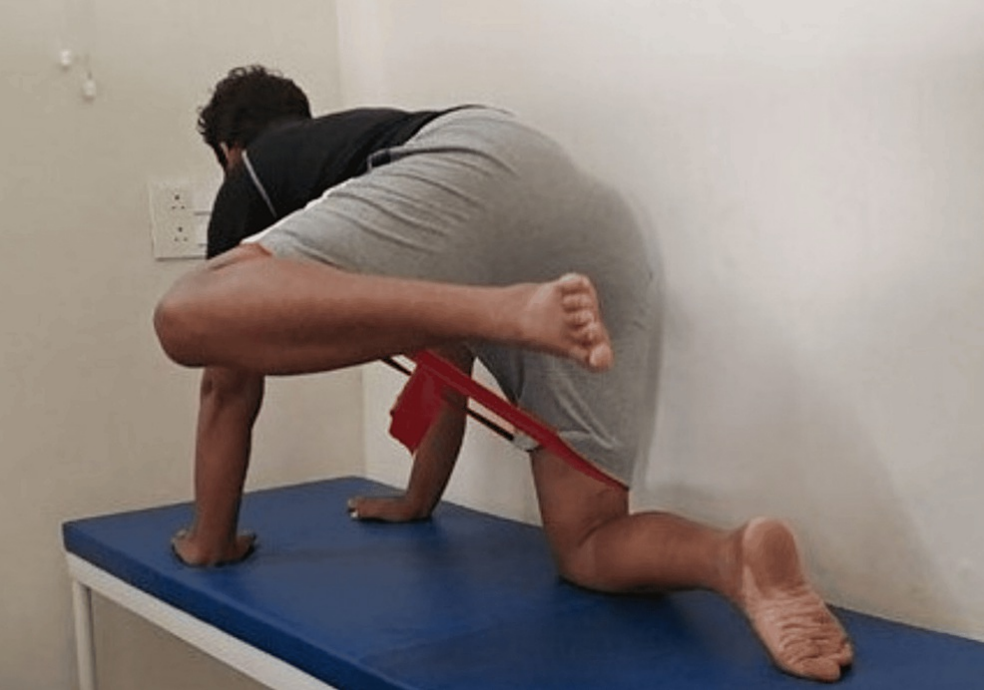
Depicting patient performing fire hydrant exercises in phase 2

**Figure 4:**
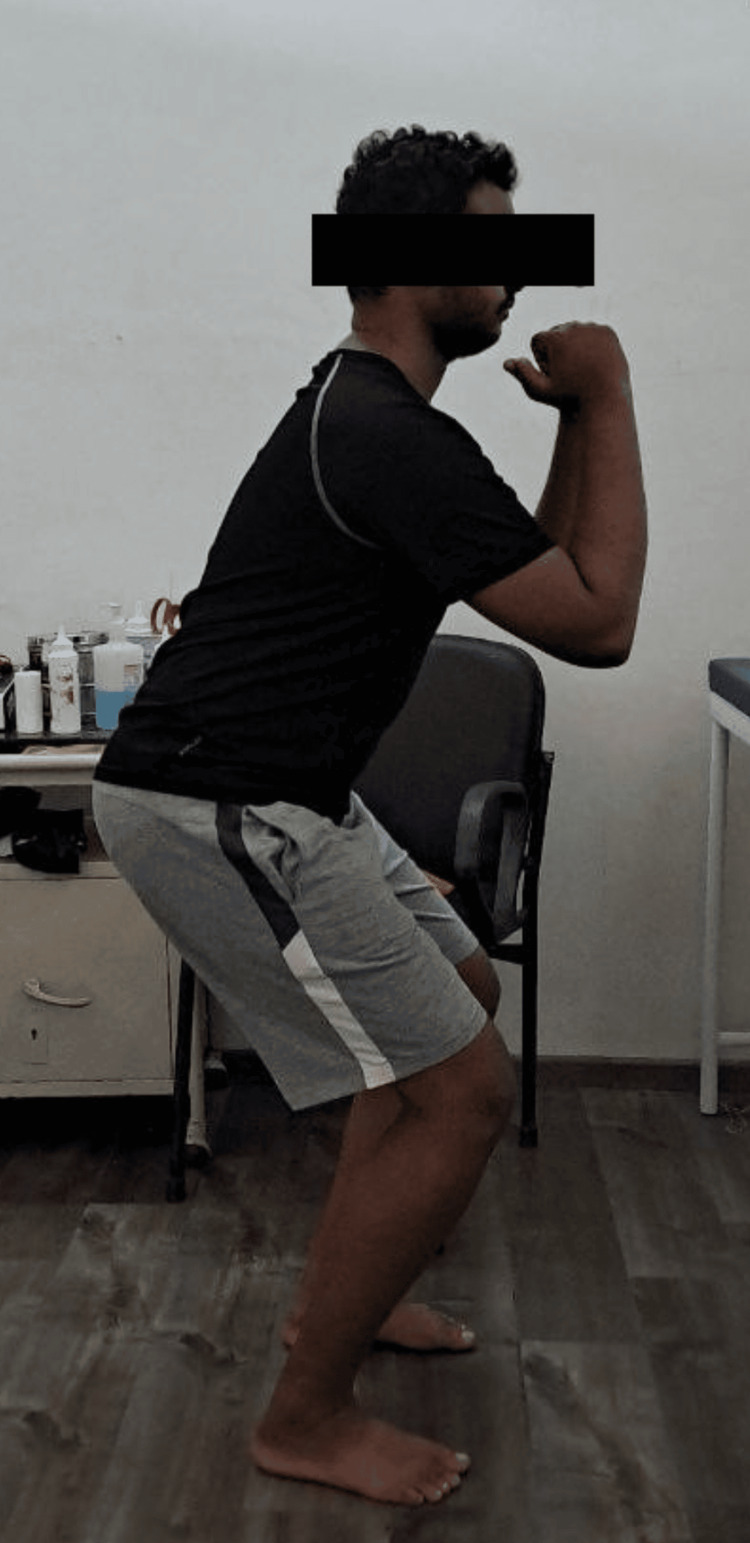
Depicts mini squats done in phase 3

**Figure 5:**
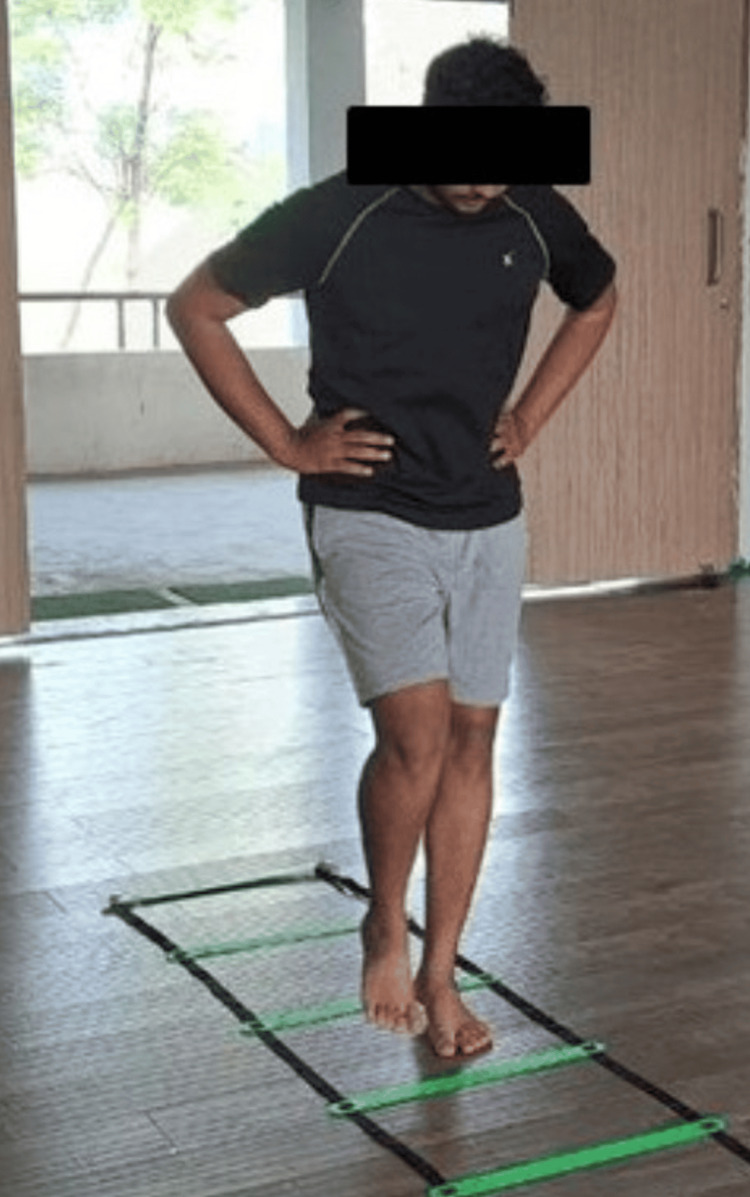
Agility ladder drill with single-leg hop

**Figure 6:**
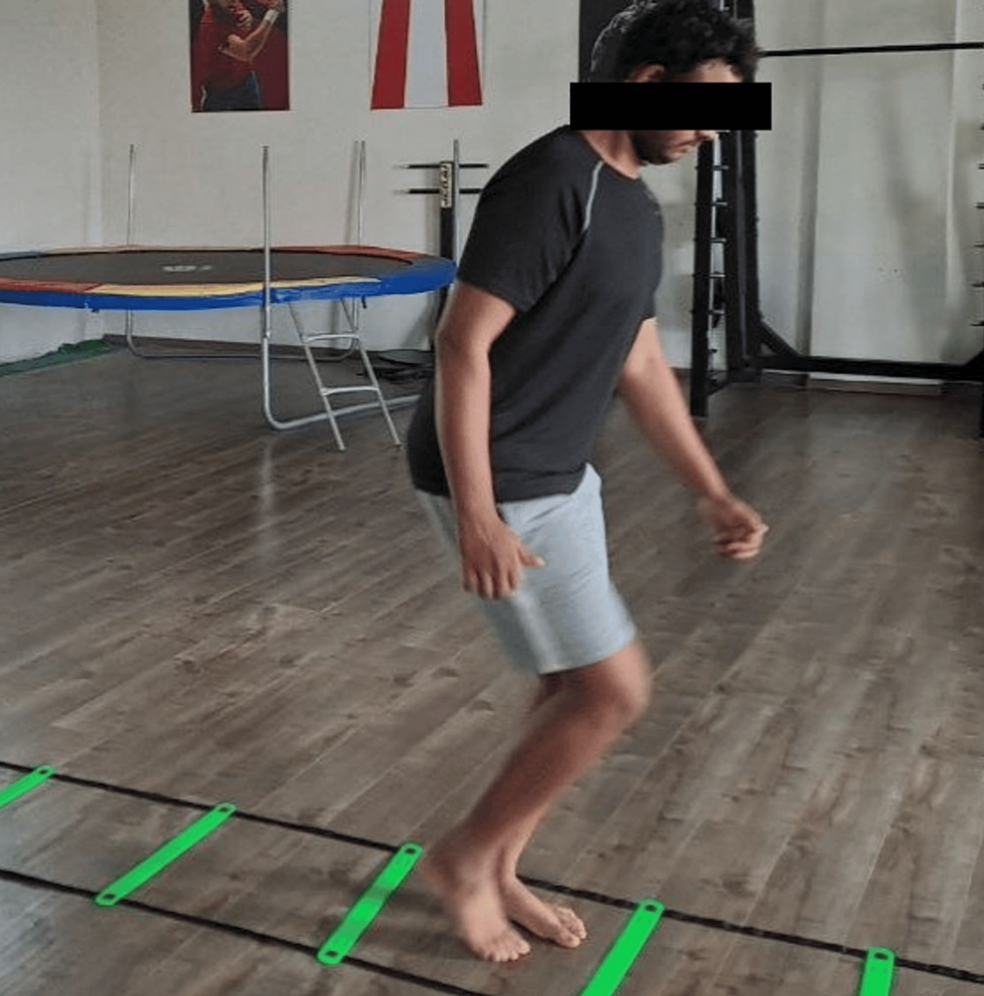
Agility ladder drill with alternate stepping only one foot in

The following figures show the exercises done in different phases. Figure 3 depicts fire hydrants being performed in phase 2; Figure 4 depicts mini squats being done in phase 3. Figure 5 and Figure 6 show various agility ladder drills being done in phase 3.

Outcome measures

The outcome measures analyzed are numeric pain rating scale (NPRS) and lower extremity functional scale (LEFS). The NPRS and LEFS scores pre-rehabilitation were seven and 18, respectively. The post-rehabilitation scores of NPRS and LEFS were 1 and 72, showing substantial progress in the outcomes, as shown in Table 4.

**Table 4:** Outcome measures scores NPRS: It consists of an 11-point scale. 0: no pain at all; 1-3: mild pain, barely noticeable, does not interfere with daily activities; 4-6: moderate pain, noticeable and bothersome, may slightly interfere with daily activities; 7-9: severe pain, difficult to ignore, significantly interferes with daily activities, 10: worst pain imaginable, unbearable. LEFS: the maximum possible score is 80 points, indicating a very high function, and the minimum possible score is 0 points, indicating a very low function. LEFS, lower extremity functional scale; NPRS, numeric pain rating scale

Outcome measures	Pre-rehabilitation (before phase 2)	Mid-rehabilitation (Post-phase 2)	Post-rehabilitation (Post-phase 3)
NPRS	7	5	1-0
LEFS	18	39	72

## Discussion

As mentioned in progress on the outcome measure, the patient demonstrated expected progress in achieving goals related to ROM, strength, gait, balance, and posture based on the prescribed protocol. Early emphasis on pain relief, joint mobilization, ROM exercises, and quadriceps activation yielded fast increases in ROM and better control and stability of the quadriceps muscles. This program incorporated early agility drills, such as ladder exercises, from the outset. This early introduction of agility training could be a key factor in accelerating recovery compared to standard protocols for MPFL reconstruction surgery.

Notably, the patient achieved milestones in a comparable or shorter time frame than others undergoing the same surgery, safely unlocking the extension brace, attaining over 120° of active flexion, progressing in non-weight-bearing (NWB) exercises, initiating advanced strengthening exercises, and achieving full weight-bearing status. This progress was observed to be on par with or faster than a study conducted by Manske et al. [14]. While the patient's episode of care needed to be completed at the conclusion of data collection, certain tasks such as running, jumping, and returning to sports could not be directly compared to Manske et al. However, based on the patient's progress, the patient would have achieved similar or faster milestones in these areas. Despite Deie et al. reporting no patellar dislocations following MPFL reconstruction, caution is advised to avoid stressing the reconstructed structures [15].

This case study highlights the potential advantages of an intensive rehabilitation program, which includes agility activities early on. While Saper et al. found that many adolescent athletes may require more than eight months to restore strength for a safe return to sports, this patient met functional benchmarks more quickly [16]. This difference could be due to the inclusion of agility drills at the beginning of rehabilitation, which promotes improved control and stability sooner and in a phased manner.

This marked variability in MPFL rehabilitation protocols, as identified by Lightsey et al., underscores the critical need to address the lack of validated guidelines [17]. While recent studies suggest the benefits of accelerated rehabilitation, this research aimed to assess the online protocols offered by academic orthopedic programs. The findings showcase a significant lack of uniformity. At the same time, most protocols recommend similar approaches for bracing and initial weight-bearing; considerable variation exists in aspects like progression to full weight-bearing; ROM goals; and the inclusion and timing of specific exercises like strengthening, stretching, and proprioception. This emphasizes the need for the study's objective - to assess this variability and potentially pave the way for the development of more standardized and evidence-based rehabilitation protocols.

However, it is important to acknowledge the limitations of a single case report, and the patient's progress with activities like running and jumping could not be directly compared to Saper et al.'s study. Additionally, caution is still required to avoid stressing the reconstructed ligament. More research is needed to figure out the best way to decide when it is safe to return to sports after this surgery.

On the downside, there are areas for improvement, notably the need for adequate support for pertinent patient-reported outcomes or physical performance measures post-MPFL reconstruction. The under-utilization of advanced physical therapy technology, such as sophisticated gait and motion analysis, could have potentially enhanced outcomes and recovery efficiency, providing deeper insights into the rehabilitation process. Additionally, there needs to be more information concerning the criteria for discontinuing the use of assistive devices and the prolonged use of orthotics, decisions solely made by the operating surgeon. A notable setback is the need for a proper outcome measure for assessing long-term outcomes. Future directions should be planned to address this deficiency and also to formulate a return to sports guidelines specific to cricket.

## Conclusions

This case report serves as a valuable guide for the clinical rehabilitation of patients post-MPFL reconstruction. It provides a rationale for procedural interventions and resources for comparing recovery timelines. However, future studies should focus on identifying more suitable tests and measures, developing systematic approaches for assessing assistive devices and brace requirements, selecting exercises that reduce stress on the MPFL, and exploring the role of various technologies in the recovery process for these patients.
